# 28561 Optimization of Heart Failure Treatment Using a Novel Application Programming Interface (API)

**DOI:** 10.1017/cts.2021.532

**Published:** 2021-03-30

**Authors:** Anthony Mack, David Cordwin, Michael Dorsch

**Affiliations:** University of Michigan College of Pharmacy

## Abstract

ABSTRACT IMPACT: This project will aid in the optimization of treatment for those with heart failure with a reduced ejection fraction in order to both maximize health benefits and minimize financial burdens. OBJECTIVES/GOALS: To evaluate the accuracy and clinical applicability of a novel web-based application programming interface in the optimization of care for patients being treated for heart failure with reduced ejection fraction (HFrEF). The purpose of this validation is to ensure the translatability of this algorithm to a clinical setting using real-world data. METHODS/STUDY POPULATION: This study is a retrospective analysis of a previously created algorithm designed to optimize therapy for patients currently diagnosed with HFrEF. Patients that are seen for HFrEF treatment at Michigan Medicine are enrolled in a heart failure registry and were included in this study. Exceptions include those with heart transplants, LVAD, and those undergoing treatment with chronic inotropes (milrinone/dobutamine). Clinically relevant information (demographics, vital statistics, labs, and medications including dose and frequency) was taken from their respective electronic health record (EHR) and this data was used as the input for the algorithm. The therapy recommendations were collected and manually compared to the 2017 ACC/AHA/HFSA guidelines to verify the accuracy of the algorithm outputs. RESULTS/ANTICIPATED RESULTS: Data is currently being collected and analyzed. At first glance, our algorithm has been successful at detecting patients that are good candidates for therapy optimization. Based on inputs given, the treatment recommendations have been appropriate when compared to the most up-to-date HF treatment guidelines. The algorithm has also correctly identified levels of urgency for therapeutic recommendations. Finally, we have also shown the algorithm to have effectiveness for identifying areas of inappropriately adjusted therapy. Preliminary results have led to changes to the functionality of the algorithm, including how medications are retrieved from the EHR’s and how medication doses are identified. Previous iterations created discrepancies in dosing and the algorithm has since been adjusted. DISCUSSION/SIGNIFICANCE OF FINDINGS: By verifying its validity, our algorithm can accurately flag patients with HFrEF that are eligible for therapy optimization and give providers the opportunity to make appropriate changes. Given the high health and financial burdens of HFrEF, our algorithm has the ability to provide significant morbidity, mortality, and financial benefits.

